# Errors in search strategies used in systematic reviews and their effects on information retrieval

**DOI:** 10.5195/jmla.2019.567

**Published:** 2019-04-01

**Authors:** José Antonio Salvador-Oliván, Gonzalo Marco-Cuenca, Rosario Arquero-Avilés

**Affiliations:** Professor, Department of Library and Information Science and Faculty of Medicine, University of Zaragoza, Zaragoza, Spain, jaso@unizar.es; Professor, Department of Library and Information Science and Faculty of Medicine, University of Zaragoza, Zaragoza, Spain, gmarco@unizar.es; Professor, Department of Library and Information Science, Complutense University of Madrid, Madrid, Spain, carquero@ucm.es

## Abstract

**Objectives:**

Errors in search strategies negatively affect the quality and validity of systematic reviews. The primary objective of this study was to evaluate searches performed in MEDLINE/PubMed to identify errors and determine their effects on information retrieval.

**Methods:**

A PubMed search was conducted using the systematic review filter to identify articles that were published in January of 2018. Systematic reviews or meta-analyses were selected from a systematic search for literature containing reproducible and explicit search strategies in MEDLINE/PubMed. Data were extracted from these studies related to ten types of errors and to the terms and phrases search modes.

**Results:**

The study included 137 systematic reviews in which the number of search strategies containing some type of error was very high (92.7%). Errors that affected recall were the most frequent (78.1%), and the most common search errors involved missing terms in both natural language and controlled language and those related to Medical Subject Headings (MeSH) search terms and the non-retrieval of their more specific terms.

**Conclusions:**

To improve the quality of searches and avoid errors, it is essential to plan the search strategy carefully, which includes consulting the MeSH database to identify the concepts and choose all appropriate terms, both descriptors and synonyms, and combining search techniques in the free-text and controlled-language fields, truncating the terms appropriately to retrieve all their variants.

## INTRODUCTION

The search for information is a basic component of systematic reviews [1, 2]. The objective of the search is to retrieve all publications that are potentially relevant to the object of study to minimize bias in forming conclusions [3]. To achieve this goal, it is essential to search multiple databases using a comprehensive search strategy that is free from errors.

Search results are evaluated primarily by two measures: recall and precision. The ideal result has high rates of both recall and precision, although this goal is difficult to achieve due to the tradeoff relationship that exists between the two [4]. Hence, it is necessary to find an equilibrium between them. Obtaining a high recall rate [3, 5] with a reasonable level of precision that minimizes the time and resources necessary to examine the retrieved records is a priority in systematic reviews.

Although the literature indicates that directly measuring whether a search for information has retrieved all the relevant records is impossible [6] and that certain factors external to the search engine can prevent finding all pertinent records (e.g., incorrect indexing [7], the lack of standardization of article abstracts or inconsistent terminology [8]), an error-free strategy can increase the recall of relevant studies and, hence, the quality of a review [9, 10].

Excellent literature is available on constructing a good search strategy, ranging from classic books in the field of documentation science [11–14] to specific manuals on systematic reviews [1, 3, 15]. To improve search quality, the following approaches have been proposed: (a) the participation of librarians and information professionals in teams who, as search experts, develop strategic reviews [1, 16–19], an approach that has been associated with higher quality search strategies [20, 21] and fewer errors [22], and (b) peer review using the standardized Peer Review of Electronic Search Strategies (PRESS) instrument [23–25].

Although numerous articles have been published regarding the quality of search strategies in systematic reviews, almost all of them have focused on determining whether these reports included complete and precise information to allow them to be reproduced [26–33]. Of reviews in the Cochrane Library, the authors found one study by Sampson and McGowan that evaluated the errors in search strategies using MEDLINE (Ovid) [9].

MEDLINE is the medical database that is most frequently used in systematic reviews to find information [34, 35] and is accessible via different interfaces. Various studies have confirmed that PubMed is the interface that authors most frequently use [36, 37]. This interface has advantages such as free access, slightly greater sensitivity than MEDLINE-Ovid in searches for systematic reviews [38], status as the most current database [20], and information from sources other than MEDLINE, such as online books and articles from life sciences journals, making PubMed the preferred option for conducting literature searches. However, to date, no studies have evaluated search errors in studies using MEDLINE/PubMed. Given that such errors are related to the characteristics and functionalities of the database and its retrieval language syntax, we propose to fill this gap in the literature in this study. Our main objective is to evaluate the search strategies of systematic reviews in PubMed to identify errors, analyze their impact on information retrieval, and propose solutions.

## METHOD

### Identification of studies

MEDLINE/PubMed was searched on February 1, 2018, using the systematic review thematic filter as a search phrase [39]. The following search statement was used with no language limitation:

Systematic [sb] Filters: Free full text; Publication date from 2018/01/01

### Selection criteria

Three inclusion criteria were defined and applied in successive phases:

1. The included articles were required to be systematic reviews, systematic review protocols, or meta-analyses that included a systematic literature database search. All other types of articles were excluded, including those that applied meta-analysis techniques using numerical data obtained from data banks rather than through information searches in bibliographic databases.
2. Only articles that used MEDLINE/PubMed for their searches were included. All publications that did not search this database or that searched MEDLINE but from which the interface could not be determined were excluded.
3. The included articles were required to include a search strategy that had been described in sufficient detail to be reproducible, and the strategy must have been explicitly described in the full article text or in supplementary files stored in MEDLINE/PubMed. Articles that did not thoroughly describe their search strategies or that pertained to other databases were excluded. Also excluded were those strategies that simply mentioned the search concepts or simply presented a sequence of search terms, whether combined with Boolean operators or not (without parentheses), indicating that such searches had been applied to all the consulted databases, but without a specific syntax or an expressed declaration that the search had been conducted through PubMed.

The three authors independently examined the abstracts and full texts of the articles selected for inclusion. Discrepancies were resolved by consensus.

### Data extraction

For each review, the search strategy obtained from the methods section and/or online supplementary files was downloaded. Based on prior studies [9, 23, 25] and our knowledge and experience, the errors that affected recall or precision were selected. Then, the meaning of some of these errors was adapted to the PubMed syntax, resulting in the following list of errors for evaluation:

- incorrect use of Boolean operators (e.g., using AND instead of OR or vice versa)
- lack of parentheses (e.g., unmatched parentheses or inappropriately combined terms due to missing parentheses)
- lack of morphological variations of the terms (e.g., not truncated, truncated but with too much specificity, or syntax errors in truncation)
- missing Medical Subject Headings (MeSH) terms (e.g., where adequate descriptors for various concepts existed in the controlled vocabulary but were not present in the search strategy)
- MeSH terms not searched in the [mesh] field (e.g., where MeSH terms are included in the strategy but are searched only in the free-text fields)
- nonexplosion of MeSH terms (e.g., where records containing more specific terms were not retrieved); no errors were considered when the MeSH terms were deliberately not exploded [mesh:noexp] or were only searched in the title fields because we assumed that the authors sought a high precision rate; however, if authors searched without field tags, truncating the terms or in [all] [tw] tags, we assumed that they wanted to achieve a high recall rate and it was considered an error
- MeSH terms not searched in free-text fields (e.g., records containing the search language terms were not retrieved from free-text fields)
- missing synonyms
- repetition of morphological variations of the terms
- term redundancy (e.g., when a phrase exists that already contains a term is searched, but the term is also searched as an OR term, or when an already included field is searched in all of the OR fields [all fields])

Information was also gathered regarding the search method that had been used for:

- phrases: double quotes, truncation, field codes, and automatic mapping
- individual terms: double quotes, truncation, field codes, and automatic mapping

To evaluate missing MeSH terms and synonyms, we consulted the MeSH controlled vocabulary. This vocabulary contains three types of terms, organized hierarchically: descriptors (main headings), qualifiers (subheadings), and supplementary concepts (supplementary concept records). We confirmed whether each term in the search strategy existed or was a MeSH term and if so, whether more specific terms and synonyms existed that appeared below the Entry Terms entry.

### Process and data analysis

A statistical analysis of the data was conducted using SPSS version 22 to obtain the frequency and percentages for each type of error.

To analyze the impact of each error type on recall, we selected a strategy from the studied reviews. A search was then performed in PubMed, first using only the fragment containing the error and then with the corrected fragment. The number of records retrieved was noted in each case. These strategies were performed on April 25, 2018.

## RESULTS

**Figure 1:**
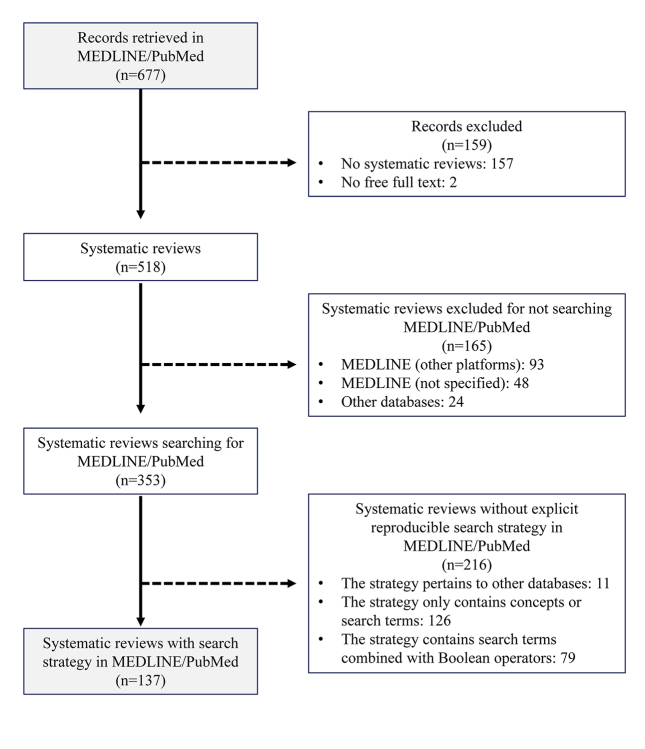
Flow chart of included studies

Our initial search retrieved 677 records. In the first phase, 159 records that were not systematic reviews were excluded: despite using the Systematic Review filter, 25 of the results consisted of data corrections, letters to the editor, editorials, and retractions; 132 were narrative reviews, controlled trials, prospective and retrospective studies, case series, and surveys; and 2 did not offer full text access. In the second phase, 165 records where MEDLINE/PubMed was not used for the search were excluded. In the third phase, all the reviews for which a specific search strategy in MEDLINE/PubMed could not be identified were excluded. Of these, 11 pertained to another database, 126 mentioned only the concepts and terms, and 79 listed only the terms combined with Boolean operators, but the strategy used in MEDLINE/PubMed was not specified. Finally, 137 systematic reviews were selected that contained a complete, reproducible search strategy for MEDLINE/PubMed (Figure 1).

### Search errors

Of the search strategies, 92.7% contained some type of error. To facilitate their presentation, the errors were grouped into 2 categories: those that affect recall and those that do not, with the former occurring more frequently (78.1%) than the latter (59.9%). Table 1 presents the frequency of the different types of errors.

**Table 1:**
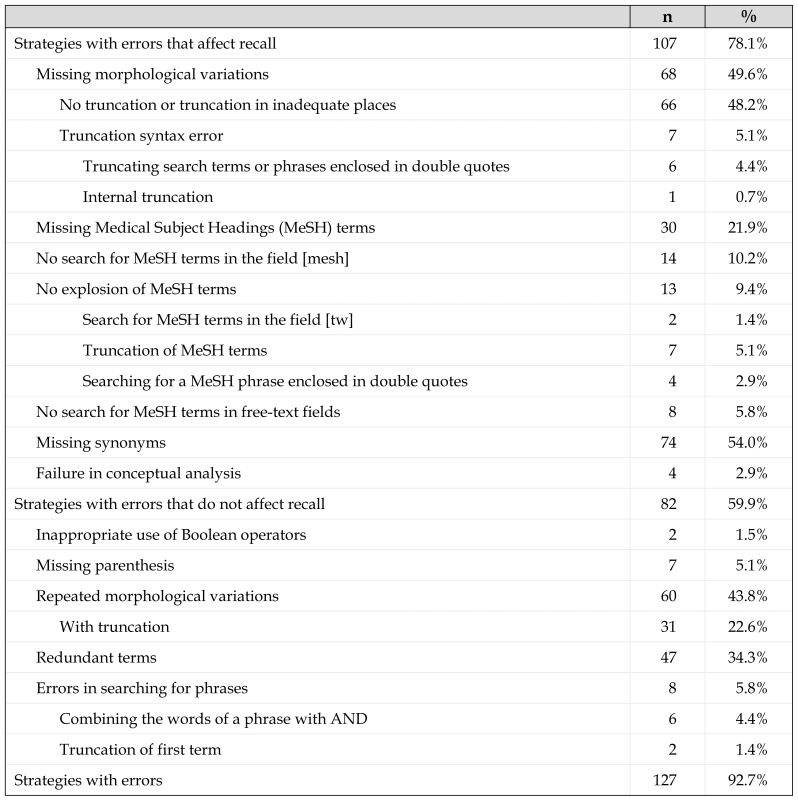
Frequency and types of errors in MEDLINE/PubMed search strategies

	n	%
Strategies with errors that affect recall	107	78.1%
Missing morphological variations	68	49.6%
No truncation or truncation in inadequate places	66	48.2%
Truncation syntax error	7	5.1%
Truncating search terms or phrases enclosed in double quotes	6	4.4%
Internal truncation	1	0.7%
Missing Medical Subject Headings (MeSH) terms	30	21.9%
No search for MeSH terms in the field [mesh]	14	10.2%
No explosion of MeSH terms	13	9.4%
Search for MeSH terms in the field [tw]	2	1.4%
Truncation of MeSH terms	7	5.1%
Searching for a MeSH phrase enclosed in double quotes	4	2.9%
No search for MeSH terms in free-text fields	8	5.8%
Missing synonyms	74	54.0%
Failure in conceptual analysis	4	2.9%
Strategies with errors that do not affect recall	82	59.9%
Inappropriate use of Boolean operators	2	1.5%
Missing parenthesis	7	5.1%
Repeated morphological variations	60	43.8%
With truncation	31	22.6%
Redundant terms	47	34.3%
Errors in searching for phrases	8	5.8%
Combining the words of a phrase with AND	6	4.4%
Truncation of first term	2	1.4%
Strategies with errors	127	92.7%

The errors that affect recall occur for two main reasons: (1) missing terms (synonyms, morphological variations, and MeSH terms) and (2) the search mode for the descriptors. Search mode errors occur because the descriptors are not searched in the [mesh] field, either explicitly or through automatic mapping; because they are not exploded (and, thus, their more specific terms are not retrieved) when searching in the text words [tw] field; because automatic mapping is disabled by truncating descriptors or enclosing MeSH phrases in double quotes; or because terms are not searched in free-text fields such as the title and abstract. During the search evaluation process, we identified additional types of errors, such as failures in the analysis of concepts.

The most frequent search errors that did not affect recall involve repetitions of morphological variations of words despite truncation and term redundancies. Neither of these errors affects information retrieval negatively with respect to either recall or precision.

Errors due to incorrect searches for a phrase lower the precision of the results by either directly combining the two terms using the AND operator or truncating the first term in a phrase formed by two or more terms separated by spaces, which disables automatic phrase search. In the latter case, PubMed combines the terms with the AND operator. Strategies that contain the Boolean operators OR and AND also yield less precision, as does failing to enclose terms in parentheses that belong to the same concept, because these strategies result in records being retrieved that do not contain all the searched concepts.

Although errors resulting from the incorrect use of Boolean operators can affect both recall and precision, of those found in our study, one has no effect when the terms are combined with the AND operator, and the other affects precision because it utilizes the OR and AND operators together without a term between them (possibly a transcription error). In this case, the second operator (the correct one) is ignored.

Truncation was used in 63 searches (46.0%). In about half of these (22.6%), variations of the terms were repeated. One-third had missing variations such that within the same search strategy, some terms were truncated, whereas others were not, and some strategies used truncation but included repeated variations.

### Search modes

There are two ways to search terms in PubMed: without field tags, which activates automatic term mapping, or with field tags.

The number of strategies that used field tags for all the terms was greater (46.7%) than the number of strategies in which all terms appeared without tags (21.2%) (Table 2). Note that searching all fields [all] is equivalent to automatic mapping, unless the terms are truncated or enclosed in double quotes, because they turn off automatic mapping. Of the strategies that use only field tags, the most frequent are those that combine searching in the MeSH field and in the title/abstract. Those strategies that searched only in the title and abstract fields are candidates for reduced recall when the terms are descriptors, as are those that search only MeSH fields, because they will fail to retrieve records that contain the search terms in the title and abstract fields.

**Table 2:**
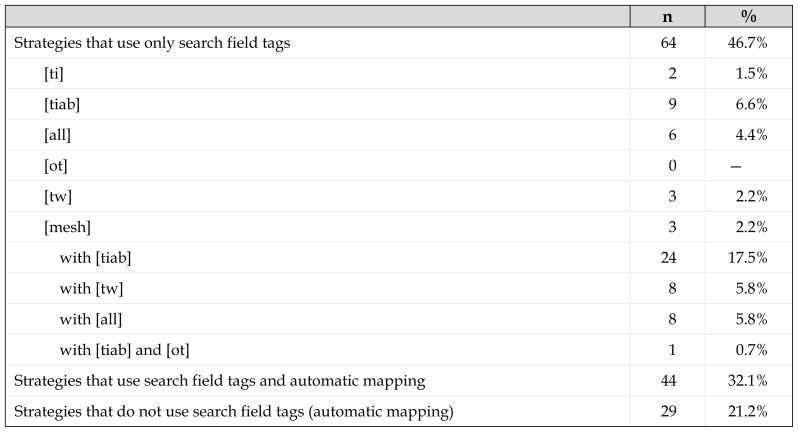
Search strategies that use field codes and/or automatic mapping

	n	%
Strategies that use only search field tags	64	46.7%
[ti]	2	1.5%
[tiab]	9	6.6%
[all]	6	4.4%
[ot]	0	—
[tw]	3	2.2%
[mesh]	3	2.2%
with [tiab]	24	17.5%
with [tw]	8	5.8%
with [all]	8	5.8%
with [tiab] and [ot]	1	0.7%
Strategies that use search field tags and automatic mapping	44	32.1%
Strategies that do not use search field tags (automatic mapping)	29	21.2%

#### Phrase searches

Phrases are searched to retrieve records that contain adjacent terms in the order indicated; this approach ensures the precision of the results. The most common approach is to use field tags. The option to search for a phrase with the terms separated by spaces is not suitable because PubMed processes them with automatic mapping, retrieving not only all records that contain the phrase (if it recognizes it as one), but also those that contain both terms but not as a phrase (combined with AND), causing noise in the results due to contextual ambiguity (Table 3).

**Table 3:**
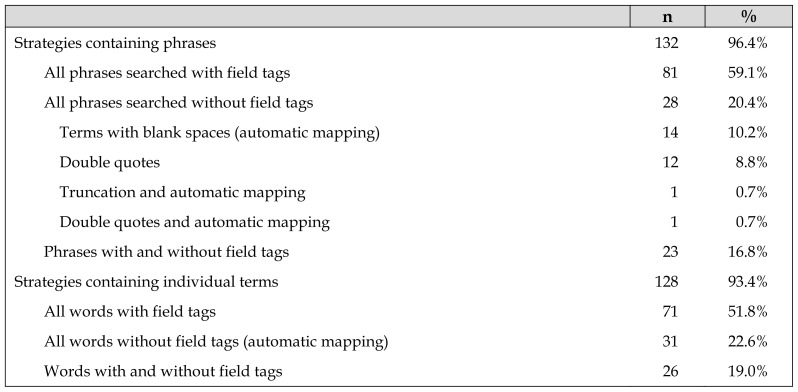
Ways to search for phrases and individual terms

	n	%
Strategies containing phrases	132	96.4%
All phrases searched with field tags	81	59.1%
All phrases searched without field tags	28	20.4%
Terms with blank spaces (automatic mapping)	14	10.2%
Double quotes	12	8.8%
Truncation and automatic mapping	1	0.7%
Double quotes and automatic mapping	1	0.7%
Phrases with and without field tags	23	16.8%
Strategies containing individual terms	128	93.4%
All words with field tags	71	51.8%
All words without field tags (automatic mapping)	31	22.6%
Words with and without field tags	26	19.0%

#### Searches for individual terms

Failure to use field tags can affect precision because PubMed may substitute the search terms with others that have different meanings; hence, it is advisable to ensure that mapping is performed in the manner desired (Table 3).

### Effects of errors on information retrieval and solutions

The tables that show the effects of errors on information retrieval are included in the supplemental appendix. Both tables include specific examples of the different error types identified in search strategies and corresponding solutions. To better differentiate between the results, the numbers of records retrieved in PubMed are shown separately for the case of errors that affect recall (Table 4, supplemental appendix) and those that do not affect recall (Table 5, supplemental appendix).

## DISCUSSION

The results of this study reveal that the percentage of search strategies that contain various types of errors is quite high (92.7%) and that 78.1% of these errors affect recall. Therefore, these errors can influence the conclusions of systematic reviews.

We found only one study (Sampson and McGowan [9]) that identified errors in search strategies. This study differed from ours in ways that should be considered when comparing them: their strategies were carried out in MEDLINE but on a different platform (Ovid), and their descriptions and number of errors did not completely agree with the results of our study. For example, our study did not include errors such as whether the search strategy was adapted to other databases, errors whose determination was subjective (term relevance), or any errors specific to the Ovid interface. Meanwhile, Sampson and McGowan did not include some errors that were analyzed in this study, such as a lack of synonyms or searches for descriptors in free-text fields.

Despite these differences, the percentages of strategies that contain at least 1 error were very similar in both studies (92.7% vs. 90.5%). We found fewer errors due to inappropriate use of Boolean operators (1.5% vs. 19.0%); however, we found more frequent errors due to missing term variations (48.2% vs. 20.6%) and redundancy (34.3% vs. 12.7%). One plausible reason was that the reviews that Sampson and McGowan analyzed were published in the Cochrane Database of Systematic Reviews, which is considered the gold standard for evidence-based practice [40]. Consequently, one might expect fewer errors in these reviews than in the reviews in our study, which had been published in any free full text journal.

Many of the errors that we found revealed a lack of knowledge regarding the principles of information retrieval and/or the specific characteristics of searching in the PubMed database. While supplemental Tables 4 and 5 describe specific solutions for each error, different aspects that influence the correct design of a search strategy and that, therefore, constitute general solutions for avoiding errors are presented below.

Search strategy design always begins with an analysis of the main concepts and the choice of terms to use for each concept. Incorrect identification of the concepts is a serious error that affects search success. The MeSH database is a very useful tool for identifying concepts and choosing appropriate terms. It is recommended that controlled vocabulary and natural language terms be used [25], regardless of whether they are synonyms or alternative terms, along with their variations and different possible sequences within a phrase [41].

All possible variations of the terms must be considered. Truncation can be used to avoid having to explicitly include all possible variants in the strategy. In PubMed, the symbol for truncation is the asterisk (*), and its effect is to retrieve all the words that contain the root (the part of the word preceding the asterisk), thus increasing recall.

Familiarity with some of the aspects of the correct use of truncation in PubMed is required:

- Only the end of a word can be truncated. Truncation to the left or within a word is not allowed.
- Descriptors cannot be truncated when searching in the [mesh] field. When descriptors are truncated, variations are not retrieved, because only the exact descriptor will be searched. For example, hypertens* [mesh] is equivalent to searching for hypertension [mesh].
- When searching for a phrase, only the last term should be truncated. If a prior term is truncated, the entry will not be searched as a phrase; instead, PubMed will search all variations of that term linked with AND to the next term.
- Truncating a term or phrase enclosed in double quotes has no effect. PubMed will retrieve all the records that contain the exact character string located before the asterisk but will not retrieve records with any of its variations.
- Truncation disables automatic mapping.

After selecting the terms, it is necessary to use search techniques with controlled language and free text. Previous studies show that the best results are obtained by combining the techniques of free text and controlled language search [40]. A search conducted exclusively with the latter can miss relevant information due to indexing failures (not all the main concepts addressed in the articles appear as descriptors) and due to the possible lack of suitable descriptors for representing a concept. Additionally, this loss is greater in PubMed because most current records do not yet have MeSH terms assigned; hence, these records would not be retrieved.

The search with controlled language consists of searching for descriptors in the [mesh] field, and it offers two advantages regarding information retrieval:

1. It improves recall. When the indexing is consistent, using a single term to search for a concept favors retrieval of all documents that address that concept without having to use any synonym because PubMed explodes the term by default—that is, it retrieves all the more specific terms located below it in the hierarchical MeSH structure.
2. It improves precision. When a term is present in the [mesh] field, it means that the concept represented by that term is addressed in a significant way in the article—much more so than if the term appeared only in the abstract.

The free-text search involves searching for natural language terms in text fields such as the title and abstract [22].

It is also important to be knowledgeable regarding the principles of information retrieval in order to avoid committing basic errors and to apply these principles to the particular characteristics of the search language of the database used. Among these, the following should be noted:

- Terms related to the same concept should be linked by the OR operator, whereas terms referring to distinct concepts should be linked with AND. When several terms are separated by spaces, PubMed processes them through automatic mapping, translating the query into phrases and/or terms combined correctly with the Boolean operators.
- The Boolean operators are processed from left to right, and the AND operator takes precedence over the OR operator. When Boolean operators with different precedence (OR, AND) exist in the same search strategy, it is important to ensure that the search order process is the desired one. Otherwise, using parentheses allows prioritization of the operations that must be executed first. For the strategy to be executed correctly, it is advisable to enclose terms that pertain to the same concept in parentheses.
- When a concept is formed by two or more terms, it is appropriate to search the terms as a phrase. The procedure for performing phrase searches in PubMed includes the following steps: (a) truncating the final term, (b) joining terms with a hyphen, (c) enclosing the phrase in double quotes, and (d) using a field tag. Failure to search as a phrase and instead combining the terms with the AND operator introduces noise due to contextual ambiguity, which increases recall but reduces precision.

An efficient search strategy design requires knowledge of the differences and similarities between both modes and between the different fields, including how such characteristics affect the recall and precision of the results. Field tags enable users to take advantage of certain search features in PubMed, such as searching for phrases and controlling the fields in which terms appear. Which field to use depends on the search objectives. In a search where a high recall rate is desirable, terms that are descriptors should be searched for in the [mesh] field and in the title and abstract fields [tiab]; terms that are not descriptors should be searched for in the title and abstract fields [tiab] and in the authors’ keywords [ot].

The fields [all] and [tw] should be used with caution with certain terms, because they can introduce noise when they appear, for example, in non-thematic fields such as author affiliation. Meanwhile, it should be taken into account that MeSH terms searched in the [tw] field are not exploded and do not retrieve more specific terms (lower recall), while terms searched in the [all] field are processed automatically unless truncated or enclosed in double quotes, in which case they are searched in all fields. However, because automatic mapping is disabled, if the terms are MeSH terms, they will not be exploded.

Failing to use field tags transfers control of the search process to PubMed’s automatic mapping, which can sometimes cause significant noise, whether due to mapping to inadequate MeSH terms and/or combining the terms in a phrase with the AND operator.

### Limitations

The study included only systematic reviews published during a single month. Nevertheless, we believe that this sample is sufficiently large to demonstrate many of the search strategy errors that occur when using PubMed.

The specific impact of the errors identified in search strategies for information retrieval was demonstrated through examples that were obtained from existing systematic reviews, and solutions were proposed based on the characteristics of PubMed. The main limitation is that the overall impact on the final result of the entire search strategy is not measured here, as doing so would have required knowing exactly what information the authors were seeking and, in many cases, modifying the complete strategy to present an error-free version, which would have made this article excessively long.

Search error identification was based on the strategies described in the selected reviews; however, it was possible that in some cases such error might have been introduced during transcription or that searchers might have utilized a version that was modified or adapted.

The effects of errors on information retrieval were demonstrated in this article using examples of search strategies on concrete topics, and this impact might be larger or smaller on other topics, according to the number of publications in the database.

Another limitation was that only systematic reviews of free full-text articles in PubMed were evaluated. Although this selection criterion did not influence the study’s main objective of identifying error types, it might have influenced the number and percentage of search strategies that generated errors because Cochrane reviews or reviews published in journals with stricter criteria were not evaluated.

## CONCLUSIONS

The importance of information searches in systematic reviews is frequently discussed in the literature. Despite this, our study reveals that the number of search strategies that contain errors is very high and that the majority of these errors affect recall. Such errors occur primarily due to the failure to use synonyms or truncations to retrieve the different morphological variants of terms. Other frequent error types (although to a lesser extent) involve missing MeSH terms and failure to retrieve more specific terms through nonexplosion.

We recommend the following measures to improve the quality of PubMed search strategies:

- Consult the controlled MeSH vocabulary: Doing so will help to identify concepts and to select adequate terms, which are two key steps for achieving success in searching.
- Combine the techniques of controlled vocabulary and free-text searches: To avoid losing current relevant information, MeSH terms should be searched in both the [mesh] field and in the title and abstract free-text fields [tiab]. Terms that are not descriptors should be searched in the [tiab] and author keyword [ot] fields.
- It is preferable to search terms and phrases using field tags rather than allowing PubMed to process the search through automatic mapping, because the more specific approach avoids PubMed mapping searches to inappropriate terms, possibly causing noise.

Terms must be truncated to retrieve all possible variations; however, it is important to consider that: (a) individual terms are truncated when they are searched in free-text fields; (b) MeSH terms are not truncated when searched in the [mesh] field; and (c) when a phrase is searched, only the last term should be truncated.

## SUPPLEMENTAL FILE

Appendix Tables 4 and 5 Examples of search errors and terms
