# Supplementary material for: Errors in search strategies used in systematic reviews and their effects on information retrieval
Source: J Med Libr Assoc. 2019 Apr 1;107(2):210–21. doi: 10.5195/jmla.2019.567 (PMC6466507; doi:10.5195/jmla.2019.567)
Supplement: Appendix [file jmla-107-210-s001.pdf]

JMLA Journal of the Medical Library Association

SC

Supplemental content to
J Med Libr Assoc. Apr;107(2):dx.doi.org/10.5195/jmla.2019.567
www.jmla.mlanet.org
© Salvador-Oliván, Marco-Cuenca, Arquero-Avilés 2019

Errors in search strategies used in systematic reviews and their effects on information retrieval

José Antonio Salvador-Oliván; Gonzalo Marco-Cuenca; Rosario Arquero-Avilés

APPENDIX

Tables 4 and 5 Examples of search errors and terms

Table 4 Search errors that affect recall: causes, effects, and solutions

Error	Cause	Solution	Article's authors	Items
Missing morphological variations				
	No truncation of terms	Truncate the terms	Al-Rifai RH, et al. [42]	
OS	infant OR child			2,531,225
CS	infant [mesh] or infant* [all] or child [mesh] or child* [all]			2,932,854
	No truncation (phrase enclosed in double quotes)	Truncate the final term to search as a phrase	Marcolino MS, et al. [43]	
OS	"decision support system"			2,731
CS	decision support system*			10,394
	Truncating too far to the right	Truncate one character to the left	Eddens L, et al. [44]	
OS	Athlete*			45,534
CS	Athlet*			75,534
	Truncation syntax error	No truncation of a term enclosed in double quotes	Ezeamama AE, et al. [45]	
OS	"educ*" [ti]			21
CS	Educ* [ti]			141,283
Missing Medical Subject Headings (MeSH) terms				
	No MeSH terms are searched in the [mesh] field	Search only the appropriate descriptors in the [mesh] and free-text fields	Haniffa R, et al. [46]	
OS	(Prognostic [mesh:noexp] OR Predictive [mesh:noexp] OR Mortality [mesh:noexp] OR Survival [mesh:noexp]) AND (Scoring system [mesh:noexp] OR Rating system [mesh:noexp])			0
CS	(prognosis [mesh:noexp] OR mortality [mesh:noexp] OR survival [mesh:noexp] OR prognos* [ti] OR predictive [ti] OR mortality [ti] or survival [ti]) AND (scoring system* [ti] OR rating system* [ti])			1,191

JMLA Journal of the Medical Library Association

Supplemental content to
J Med Libr Assoc. Apr;107(2):dx.doi.org/10.5195/jmla.2019.567
www.jmla.mlanet.org
© Salvador-Oliván, Marco-Cuenca, Arquero-Avilés 2019

Table 4 Search errors that affect recall: causes, effects, and solutions (continued)

Error	Cause	Solution	Article's authors	Items
	A phrase that contains two concepts for which MeSH terms exist is searched	Perform a correct conceptual analysis: search the two concepts separately in the [mesh] and [tiab] fields	Johannsen M, et al. [47]	
OS	"post mastectomy pain syndrome" [tiab]			24
CS	(Mastectomy [mesh] OR mastectomy* [tiab]) AND (pain postoperative [mesh] OR postoperative pain [tiab])			522
C. MeSH terms not searched in the [mesh] field				
	The descriptors are searched only in the [tiab] field	Search the descriptors in the [mesh] field as well	Cabrini L, et al. [48]	
OS	intubation [tiab]			41,482
CS	intubation [tiab] OR intubation [mesh]			72,315
D. No explosion of MeSH terms				
	Searching for MeSH phrases enclosed in double quotes, with or without field tags [all]: disable automatic mapping	Search the MeSH phrases in the [mesh] field	Britt-Spells AM, et al. [49]	
OS	"social discrimination"			1,681
CS	"social discrimination"[mesh] OR "social discrimination"[all]			4,724
	Searching for MeSH terms in the [tw] field: Disable automatic mapping	Search the MeSH terms in the [mesh] field	Ezeamama AE, et al. [45]	
OS	learning [tw] OR cognit* [tw]			638,019
CS	learning [tw] OR cognit* [tw] OR learning [mesh] OR cognition [mesh]			828,994
	Truncating MeSH terms: disable automatic mapping	Search the MeSH term without truncation in the [mesh] field and in all the fields with truncation	Gadalla MA, et al. [50]	
OS	Ultraso* [all]			456,580
CS	Ultraso* [all] OR ultrasonography [mesh]			575,661

JMLA Journal of the Medical Library Association

SC
Supplemental content to
J Med Libr Assoc. Apr;107(2):dx.doi.org/10.5195/jmla.2019.567
www.jmla.mlanet.org
© Salvador-Oliván, Marco-Cuenca, Arquero-Avilés 2019

Table 4 Search errors that affect recall: causes, effects, and solutions (continued)

Error	Cause	Solution	Article's authors	Items
MeSH terms not searched in the free-text fields				
	MeSH terms are only searched in the free-text fields	Search also the MeSH terms in the [ti] (higher precision) or [tiab] (higher recall) field	Babu GR, et al. [51]	
OS	Obesity [mesh] AND hypertension [mesh] AND (prevalence [mesh] OR incidence [mesh])			2,828
CS1	Obes* [ti] AND hypertens* [ti] AND (prevalence [ti] OR incidence [ti])			150
	#2 not #1			59
CS2	(obes* [tiab] OR obesity [mesh]) AND (hypertens* [tiab] OR hypertension [mesh]) AND (prevalence [tiab] OR prevalence [tiab] OR incidence [tiab] OR incidence [mesh])			12,823
Missing synonyms				
	No synonym is searched	Search all possible synonyms in the free-text fields (and MeSH terms in the [tiab] field)	Jiang YQ, et al. [52]	
OS	"Atrial Fibrillation"[Mesh]			46,134
CS	atrial fibrillation [mesh] OR atrial fibrillation* [tiab] OR auricular fibrillation* [tiab]			68,419
	The synonyms are searched in the [mesh] field	Search the synonyms in the free-text fields	Buczinski S, et al. [53]	
OS	(refractometry[Mesh] OR index, refractive[Mesh] OR indices, refractive[Mesh])			7,618
CS	refractometry[mesh] OR refractometry [tiab] OR refractive index [tiab] OR refractive indices [tiab]			20,608
Failure in conceptual analysis				
	Two different concepts are searched as a phrase	Search for the two concepts combined by AND	Nakhjavan-Shahraki B, et al [54]	
OS	"schwann cells of the olfactory nerve"[tiab] OR "olfactory schwann cell*"[tiab]			0
CS	(schwann cells [mesh] OR schwann cell* [tiab]) AND (olfactory nerve [mesh] OR olfactory nerve [tiab] OR olfactory bulb [mesh] OR olfactory bulb [tiab])			307

OS=original strategy; CS=corrected strategy.

JMLA Journal of the Medical Library Association

SC
Supplemental content to
J Med Libr Assoc. Apr;107(2):dx.doi.org/10.5195/jmla.2019.567
www.jmla.mlanet.org
© Salvador-Oliván, Marco-Cuenca, Arquero-Avilés 2019

Table 5 Search errors that do not affect recall: causes, effects and solutions

Error	Cause	Solution	Article's authors	Items
Errors in Boolean operators				
	Use AND instead of OR (or vice versa)	Combine terms related to the same concept with OR: combine terms that refer to different concepts with AND	Chiarito M, et al. [55]	
OS	(mitraclip[All Fields] AND ("mitraclip"[Mesh Terms] OR "mitraclip"[All Fields]))			779
PT	mitraclip[All Fields] AND "mitraclip"[All Fields]			
CS	Mitraclip [all fields]			779
Missing parentheses				
	Not enclosing terms related to the same concept in parentheses (and truncating the first term in a phrase)	Enclose terms pertaining to the same concept in parentheses (when searching for a phrase, only the final term should be truncated)	Hummel P, et al. [56]	
OS	fall* [Title/abstract] AND communit* [Title/abstract] AND RCT [Title/abstract] OR randomi* controlled trial [Title/abstract]			98,895
CS	fall* [Title/abstract] AND communit* [Title/abstract] AND (RCT [Title/abstract] OR randomized controlled trial [Title/abstract])			287
Redundancy of terms				
	Terms or phrases are repeated unnecessarily (phrases including a searched single term)	Do not search for phrases containing one of the terms searched for individually	Bos M, et al. [57]	
OS	"massive chronic intervillositis"[tw] OR "chronic intervillositis"[tw] OR "chronic histiocytic intervillositis"[tw] OR "histiocytic intervillositis"[tw] OR "intervillositis"[tw]			73
CS	Intervillositis [tw]			73
Repetition of morphological variations				
	With truncation (lack of knowledge of the existence or utility of truncation)	Know that truncating a term retrieves the variations that exist before the symbol (*)	De Boer J, et al. [58]	
OS	schizophren*[tiab] OR schizophreniform[tiab]			114,659
CS	schizophren*[tiab]			114,659
	Without truncation	Use the truncation	De Groot AF, et al. [59]	
OS	"RANKL inhibitor"[tiab] OR "RANKL inhibitors"[tiab] OR "RANKL inhibition"[tiab] OR "rankl inhibiting"[tiab]			193
CS	Rankl inhibit*[tiab]			193

Supplemental content to
J Med Libr Assoc. Apr;107(2):dx.doi.org/10.5195/jmla.2019.567
www.jmla.mlanet.org
© Salvador-Oliván, Marco-Cuenca, Arquero-Avilés 2019

Table 5 Search errors that do not affect recall: causes, effects and solutions (continued)

Error	Cause	Solution	Article's authors	Items
Errors in searching for phrases				
	The terms of a phrase are combined with the AND operator	A concept formed by two or more terms should be searched as a phrase (in free-text and/or [mesh] fields if it is a descriptor)	Adam S, et al. [60]	
OS	(prostate[Title/Abstract] OR prostatic[Title/Abstract]) AND (cancer[Title/Abstract] OR adenocarcinoma[Title/Abstract] OR adenocarcinomas[Title/Abstract] OR carcinoma[Title/Abstract] OR carcinomas[Title/Abstract] OR neoplasia[Title/Abstract] OR neoplasm[Title/Abstract] OR neoplasms[Title/Abstract])			134,159
CS1	prostate cancer [tiab] OR prostate adenocarcinoma* [tiab] OR prostate carcinoma* [tiab] OR prostate neoplas* [tiab] OR prostatic cancer [tiab] OR prostatic adenocarcinoma* [tiab] OR prostatic carcinoma* [tiab] OR prostatic neoplas* [tiab]			115,364
CS2	prostate cancer [tiab] OR prostate adenocarcinoma* [tiab] OR prostate carcinoma* [tiab] OR prostate neoplas* [tiab] OR prostatic cancer [tiab] OR prostatic adenocarcinoma* [tiab] OR prostatic carcinoma* [tiab] OR prostatic neoplas* [tiab] OR prostatic neoplasms [mesh]			141,351
	Truncation of the first term in a phrase (causing PubMed to combine them with the AND operator)	Search for the phrase, repeating possible variations of the first term (truncate only the final term in a phrase)	Boatin AA, et al. [61]	
OS	(Robson* classification*)			366
PT	(robson[All Fields] OR robson's[All Fields] OR…) AND (classification[All Fields] OR classification'[All Fields] OR…….)			
CS	Robson classification* [all] OR robson's classification* [all]			73
Errors caused by automatic mapping				
	Incorrect translation of the MeSH term into other terms that generate noise	Ensure that automatic mapping translates the MeSH term appropriately, or use field labels	Chen H, et al. [62]	
OS	Android			3,455
PT	"methyltestosterone"[Mesh Terms] OR "methyltestosterone"[All Fields] OR "android"[All Fields]			
CS1	"android" [all]			1,732
CS2	android [tiab]			1,731

OS=original strategy; CS=corrected strategy; PT=PubMed translation.
